# Jscatter, a program for evaluation and analysis of experimental data

**DOI:** 10.1371/journal.pone.0218789

**Published:** 2019-06-24

**Authors:** Ralf Biehl

**Affiliations:** Jülich Centre for Neutron Science & Institute of Complex Systems (JCNS-1&ICS-1), Forschungszentrum Jülich, Jülich, Germany; Centre National de la Recherche Scientifique, FRANCE

## Abstract

The aim of Jscatter is the processing of experimental data and physical models with the focus to enable the user to develop/modify their own models and use them within experimental data evaluation. The basic structures *dataArray* and *dataList* contain matrix-like data of different size including attributes to store corresponding metadata. The attributes are used in fit routines as parameters allowing multidimensional attribute dependent fitting. Several modules provide models mainly applied in neutron and X- ray scattering for small angle scattering (form factors and structure factors) and inelastic neutron scattering. The intention is to provide an environment with fit routines, data handling routines (based on NumPy arrays) and a model library to allow the user to focus onto user-written models for data analysis with the benefit of convenient documentation of scientific data evaluation in a scripting environment.

## Introduction

Most computer programs used for data evaluation allow to read data files, provide models for fitting with an appropriate fit algorithm and storage of the results. Some allow inclusion of user-defined models for fitting. A less common feature is that during the fit procedure multiple datasets are fitted simultaneously taking the experimental parameters (metadata) into account. As a general example we might think about a set of dynamic light scattering experiments (DLS) measuring the intensity correlation function of colloidal particles dispersed in a solvent. This experiment can be done at various temperatures and at multiple scattering vectors that influence the measured correlation functions, which depend on solvent viscosity (respectively, on temperature) and scattering vector. To fit all data together the model may include the temperature dependent viscosity of the solvent to allow fitting of a hydrodynamic radius directly.

Jscatter implements correct usage of these experimental parameters by storing related metadata (e.g. temperature or wavevector) as attributes of a *dataArray* and automatic usage of these e.g. in a fit procedure. Complex evaluations are possible as e.g. combined fit of neutron and X-ray scattering data. Based on an open platform as Python with NumPy/SciPy[1] as basis, Jscatter allows fast reliable development of physical models also for non-experts in programming. The computation can be sped up by usage of multi core computers through the standard Python libraries multiprocessing and/or compiled code through various open projects (e.g. *f2py*, *numba*) if necessary. Usage of scripts or Jupyter Notebooks [2] allows a step-by-step development of reusable models and evaluation procedures. This allows also an easy evaluation of large datasets e.g. from timeseries and simultaneous document evaluation of experiments from raw measured data to final conclusions. In special the documentation is difficult in common GUI based programs.

Models are based on standard Python function usually defined in a script. These can be standard functions allowing longer more complicated calculations or *lambda* functions as short one-line anonymous functions with a single expression as e.g. *f = lambda x*,*a*,*b*:x*a+b. If the parameter names of the model are found in the actual fitted *dataArray*/*dataList* as attributes they are automatically used as fixed parameters. Results can be stored as human readable ASCII file. The file format allows retrieving of the data attributes without loss of information.

An extensible model library is provided which contains currently mainly models as form factors for small angle scattering (*formfactor*), fluid and crystalline structure factors (*structurefactor*) and inelastic neutron scattering models (*dynamic*). Additional modules contain vector-oriented quadrature routines and material data related to scattering length densities (*formel*). The module *sas* provides methods for smearing and desmearing (according to the Lake algorithm) of small angle scattering data (neutron and X-ray) and evaluation of 2D detector images. Beside the *Beginners Guide* the module *examples* contains more than 30 executable examples to learn Jscatter usage and build a basis for user scripts.

Jscatter is available under the terms of the GNU General Public License (GPLv3) and hosted at https://gitlab.com/biehl/jscatter. Full documentation including installation instructions, a *Beginner’s Guide* and explicit examples is hosted at http://jscatter.readthedocs.io. A set of Jupyter notebooks is included in the examples that can be run in a *mybinder*[3] live demonstration for testing/evaluation of Jscatter (see [4] for the direct link to open a *mybinder* instance). Jscatter can be installed from the Python Package Index (PyPI, https://pypi.org/project/jscatter/) repository by “*pip install jscatter*” on Linux/macOS/Windows as described in the documentation.

In the following basic usage of Jscatter with short examples will be given demonstrating the basic functionality. Then main modules are described with a more detailed description for models that are less common, unique or deviate with respect to other programs providing similar models.

## Basic usage

**Fig 1:**
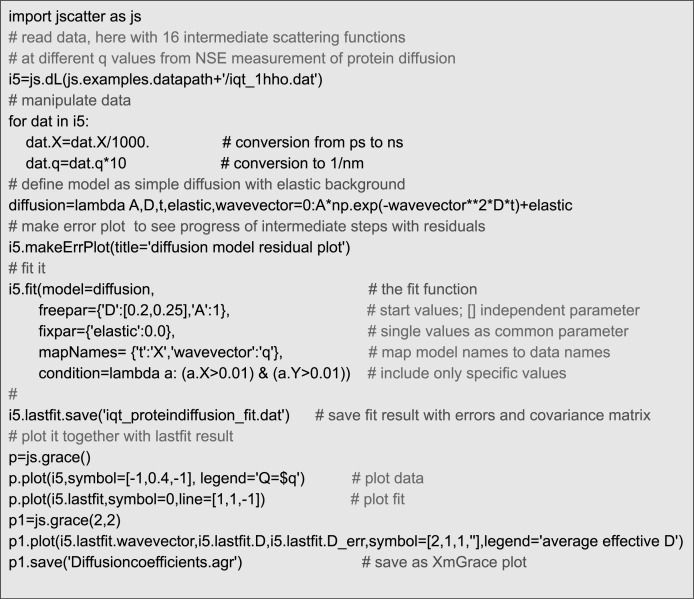
A script showing the workflow using Jscatter. The resulting plot is shown in Fig 2.

**Fig 2:**
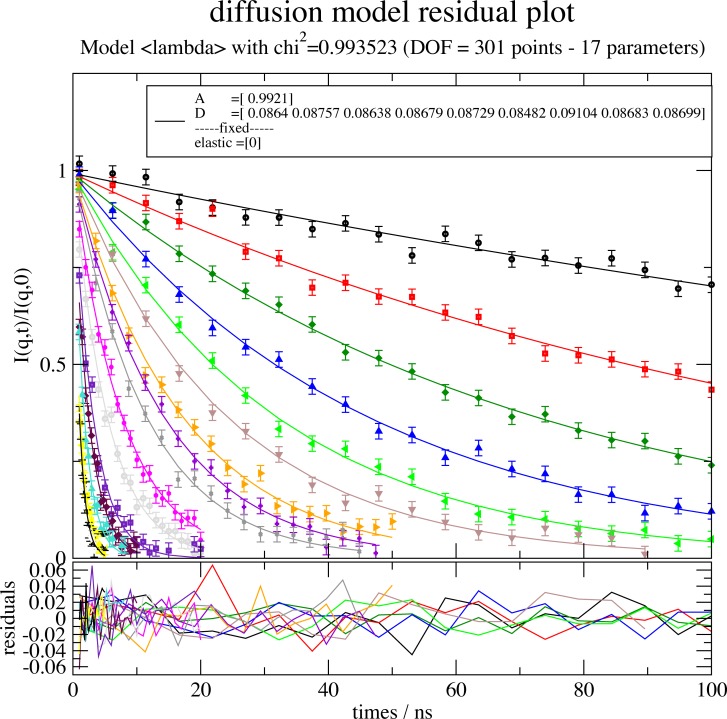
Residual plot showing data with the model calculation and respective residuals. The initial output has slightly changed adding correct axis titles, shortening and moving the legend and adjusting the scale using the Grace graphical user interface.

**Fig 3:**
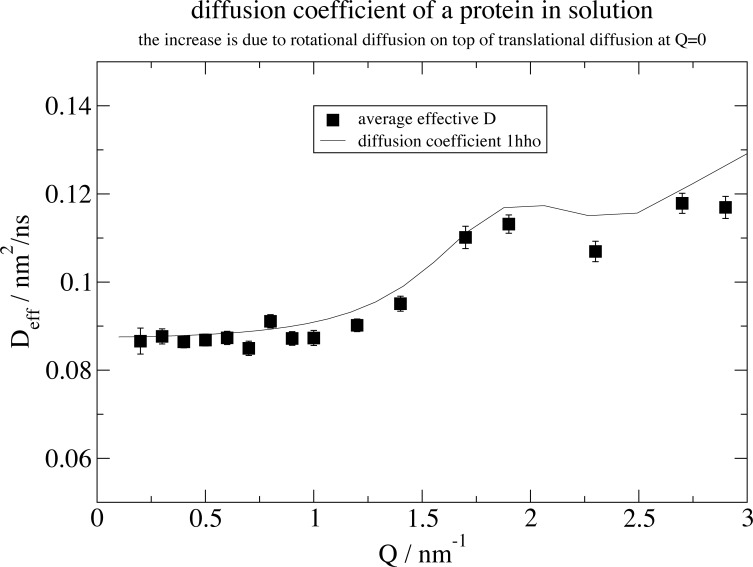
Result for the effective diffusion D_eff_(Q) together with the expectation according to a rigid protein structure. A common amplitude *A* was fitted. The upturn in diffusion is due to the additional effect of rotational diffusion if the scattering vector reaches the length scale of the protein (here hemoglobin)[5]. Axis legend and legends are inserted in Grace after plotting.

For convenience Ipython, a command shell for interactive computing with code completion, history and syntax highlighting, is recommended. Alternatively, Jupyter Notebooks can be used. A typical example for the analysis of neutron spinecho spectroscopy (NSE) measurement is shown in Fig 1. The workflow contains reading of data from a file, handling of data, defining a model function, fitting the model showing intermediate results in a residual plot (see Fig 2) and saving of the results (see Fig 3). The script can be developed step-by-step in a text editor by copy-and-paste to a Python shell. Later the script can be executed by *run scriptname*.*py*. Models can be extended or changed by changing the script and rerun it to find a suitable model describing the data. The script can be run at a later time and documents the evaluation of scientific experiments from measured experimental data up to a figure published in a scientific journal.

Model functions used during fitting are by intention defined by the user. Simple fit models can be defined in scripts or as *lambda* function in one line in an interactive command shell. Complex models may have several contributions with different physical origins or combine different experimental techniques described by a model. For instance, the example *How to build a more complex model* illustrates how to combine an ellipsoidal form factor and a structure factor including the particle density to get the scattering in absolute units. To allow maximum flexibility the modules included in Jscatter contain only basic models without backgrounds or other contributions that depend on the explicit experimental setup. In this way the user can adopt physical models to the explicit experiment and e.g. use a polynomial background fit, included a smoothed background or include parameter distributions e.g. to include particle size polydispersity. Additionally, the user can develop a user library off his own models in a Python module independent of Jscatter.

## Modules

### DataArray/DataList

The basis of Jscatter is Python with the main libraries NumPy for array related methods and SciPy for mathematical functions and the basic fit routines. Jscatter implements *dataArray* as subclass of NumPy *ndarrays*, with the ability to use attributes for storage of metadata as temperature or wavevector related to a measurement or result of a simulation. *dataList* is a subclass of *list* (part of Python base libraries) which allows to store multiple *dataArray* of different size. Together *dataArray* and *dataList* allow storage of experimental data with multiple parameters, as it is typical for measurements or simulations spanning a variety of dependent parameters. The advantage of NumPy ndarrays over other data structures is that ndarrays implement methods common to users with a basic knowledge of matrix algebra. *dataArray*/*dataList* reside in the module *dataArray* and can be accessed from the top level as *jscatter*.*d*A and *jscatter*.*dL*.

Treatment of read data stored in *dataArray* can be done by standard NumPy *ndarray* functionality accessing individual elements or slicing to access subparts of arrays as block, columns or lines. Attributes can be set to store metadata or results of computations.

Reading and interpretation of data from ASCII text files is done on basis of the first words in a line. Two numbers are interpreted as matrix-like data. A string followed by a number that can be interpreted as float is regarded as an attribute name with the remainder of the line as content. Anything else is handled as a comment, which is also stored with the *dataArray* and can be processed later if needed. Files containing multiple matrix-like datasets can be read as *dataList*. Here a new *dataArray* is started, if attributes or empty lines follow matrix-like data or a specified keyword. Reading multiple files into a single *dataList* is possible using wildcard characters (*?) or by appending a new read *dataList*. A *dataList* may contain hundreds of *dataArray* which can be later filtered according to attributes to build subsets. Options allows reading of most matrix-like ASCII text files e.g. by replacing characters/words or selecting/ignoring columns. *dataArray*`s can also be created directly from *ndarrays* as result of a simulation or from external libraries reading data formats as HDF5[6] adding corresponding attributes after *dataArray* creation. Automatic attributes as *X*, *Y*, *eY* simplify plotting routines and define the columns for fitting in multi column *dataArrays*. The column indices for up to 3 dimensions with errors can be set during reading of data or changed later. In simulation multi column *dataArray’*s allow to add intermediate output to be stored together with the final data. E.g. the scattering intensity of particles as the product of form factor F(q) and structure factor S(q) can be stored with columns [q, S(q)F(q), F(q), S(q)] for later evaluation.

Jscatter implements several fit algorithms (from *scipy*.*optimize*) as method of *dataArray/dataList* on the basis of chi-square minimization. The fastest is ‘leastsquare’, a Levenberg-Marquardt algorithm as a wrapper around MINPACK’s *lmdif* and *lmder* algorithms, compared to the other implemented methods as ‘*BFGS’* or ‘*Nelder-Mead*’[7–9]. As a method to find a global minimum a differential evolution algorithm is implemented selecting candidate solutions with stepwise improvement[10]. An extensive description of the algorithms is given in *scipy*.*optimize*.

The fit algorithms allow fitting with the ability to access data attributes as fixed parameters for each *dataArray* just by using the name of the attribute in the model. Fit parameters can be common in a *dataList* or be independent fit parameters for each *dataArray* in a *dataList* with optional limiting conditions or explicit limits. The behavior is changed by simply setting a single value or a list of values as start parameter invoking the fit process. Fitting was tested for large datasets e.g. with a series of 300 time resolved SAXS measurements and fitting of several independent and common parameters. Fit results are accessible as attribute *lastfit* including best parameter estimates, corresponding error bars, covariance matrix and other fit related quantities as e.g. model name and can be saved as *dataList*/*dataArray* in an ASCII text file.

The fit procedure accepts models defined as Python functions (including *lambda*) which return the function values as *ndarray* vector or as *dataArray* with *Y* defined. If data contain *X*, *Z*, *W* columns 2D/3D fits as e.g. for 2D image data with the function value in *Y* are possible (see help of *dataList*.*fit* for examples). If *dataArray/dataList* have defined *eY* columns these are used as 1-sigma errors to weight *Y*. After a successful fit, the model can be simulated with changed parameters to elucidate the effect of parameter variation. Models returning *dataArray’*s may include additional attributes calculated inside of the model that can be used later for evaluation as these are included in *lastfit*.

Large *dataLists* can be filtered according to attributes to select subsets (*filter*), *dataArrays* can be reduced by averaging in intervals with a linear or logarithmic separation scale (*prune*), interpolated linear, polynominal or by bispline.

### Grace/Mpl

For plotting the default is *Grace*, a free 2D graph plotting tool for Unix-like systems[11]. The module is *GracePlot* and a shortcut to open a plot is *p = jscatter*.*grace()*. *Grace* allows plotting from the command line but also adjusting the graph from a GUI interface to produce publication quality figures. Grace figures are stored in an ASCII format that can later be reused and changed. Export to usual graphic formats for publication is included. Additionally, a rudimentary interface *mpl* to *matplotlib* is included that simplifies plotting using *X*, *Y*, *eY* for a first fast draft output. Matplotlib, as a quasi-standard in plotting with Python, can be used directly and is needed for 3D plots[1].

### Formel

“Formel” is the German word for formulary. This module contains useful models or methods that may be used in the other modules or are standalone models (not justifying an additional module). Different quadrature rules as Simpson rule, adaptive Gaussian quadrature, fixed Gaussian quadrature and spherical average in vectorized form are included. Vectorized integration speeds up quadrature as NumPy compiled functions are used more efficient. Adaptive Gaussian quadrature, fixed Gaussian quadrature allow parallel computation of the integrand. The function *parDistributedAverage* computes a function with a parameter distributed by statistical distribution as ‘normal’, ‘lognorm’, ‘gamma’, ‘lorentz’, ‘uniform’, ‘poisson’, ‘duniform’. Sedimentation profiles as solutions to the Lamm-equation including and excluding the bottom equilibrium distribution can be calculated[12,13]. Material data as scattering length density, water compressibility, water dielectric constant are given. For physical constants the SciPy module constants is advised. For numerical integration Fibonacci lattices and pseudo random grids can be computed.

### Parallel

To speed up computations on a multiprocessor machine the module *parallel* offers an easy interface to the standard Python module *multiprocessing* within a single command. This provides parallel processing of a function for a list of values in case of embarrassingly parallel problems. Additionally, a function for a parallel spherical averaging using a Fibonacci lattice or a pseudorandom distribution on the sphere is implemented. For Monte Carlo Integration of new functions the pseudorandom Halton sequence is given as a choice for random samples[14].

### DLS

This module contains a wrapper around the original CONTIN algorithm for the evaluation of dynamic light scattering data. It calls the original FORTRAN code from S. Provencher[15].

### Small angle scattering

The module *smallanglescattering* (shortcut *sas*) allows smearing/desmearing of SAS data according to Pedersen[16] and for Kratky cameras as described by Lake[17]. Desmearing is implemented according to the Lake algorithm[17] with an improvement proposed by Vad introducing a smoothing and an automatic convergence criterion to stop the iterative desmearing algorithm[18]. Additional functions include silver behenate (AgBe) reference spectrum for Q calibration[19] and absolute water reference including anorganic components to calibrate the absolute scattering for SAXS[20]. To access raw data from SAXS cameras stored as TIFF files, these files can be read, masked and displayed in 2D format as *sasImage*. Basic mathematical functions can be used for evaluation in 2D as well as filters (e.g. gaussian kernel for smoothing). Calibration with AgBe allows recalibration of the detector distance, defining the beam center and radial averaging. 2D fitting of *sasImages* by 2D structure factors is demonstrated in an example.

### Form factors

The *formfactor* module (shortcut *ff*) and later mentioned *structurefactor* module contain models also available in other common small angle scattering programs like SASfit or SasView[21,22]. The scattering intensity *I*(*Q*) of *N* equal particles in a volume *V* is *I*(*Q*) = *nF*(*Q*)*S*(*Q*) with particle form factor $F(Q)=\langle F_{a}(Q),F_{a}^{*}(Q)\rangle =\langle {\left|F_{a}(Q)\right|}^{2}\rangle$, structure factor *S(Q)* and particle density *n* = *N*/*V*. 〈∙〉 indicates the ensemble average and * the complex conjugate. The single particle scattering amplitude is $F_{a}\left(Q\right)=\int_{V_{p}}b\left(r\right)e^{iqr}dr=\sum_{N}b_{i}\;e^{iqr_{i}}$ with continuous scattering length *b(r)* and particle volume *V*_*p*_ or related to discrete subparticles (atoms) with scattering length *b*_*i*_. Alternatively, for homogenous particles a normalized scattering amplitude may be defined as ${\hat{F}}_{a}\left(Q\right)=F_{a}\left(Q\right)/\int_{V_{p}}b\left(r\right)dr=F_{a}\left(Q\right)/\sum_{N}b_{i}$. This leads to the additional factor $I_{0}=V_{p}^{2}\rho_{p}^{2}$ as particle forward scattering with average scattering length density $\rho_{p}=\frac{1}{V_{p}}\int_{V_{p}}b\left(r\right)dr$. In general, the scattering length density of a solvent *ρ*_*s*_ is considered by the difference of particle scattering length density and solvent scattering length density *ρ* = *ρ*_*p*_−*ρ*_*s*_.

In the *formfactor* module the formfactor *F(Q)* is calculated to allow easier description of particles with inhomogeneous scattering length densities (e.g. multishell particles). For formfactors that don’t reference an explicit material scattering length density as for example the Beaucage formfactor, the normalized formfactor ${\hat{F}}_{a}(Q)$ is given.

Standard models as Beaucage model, generalized Guinier model, cube, superball, sphere with fuzzy surface, Teubner-Strey model, Gaussian chain, wormlike chain or ring polymers are implemented[23–30]. Standard geometrical models as sphere, ellipsoid of revolution, disc and cylinder are implemented as multishell shapes with unlimited number of shells[31]. This allows shapes with hollow core, core shell particles or gradual changing shells approximated as multiple thin shells. The cylinder model allows caps with diameter larger than the cylinder (barbell shape) or smaller (lens shape)[32,33]. For disordered multilamellar vesicles the model of Frielinghaus is used[34]. The scattering of a cylinder filled with ellipsoids is calculated in *ellipsoidFilledCylinder*[35]. To simulate polydispersity or multimodal distributions integration functions for a size parameter are given.

The scattering of arbitrary shaped particles can be calculated by *cloudScattering*. The desired shape is represented by a cloud of subparticles representing the desired shape as a kind of volume integration. The subparticle itself may be described by a subparticle formfactor *b*_*i*_*(q)* as sphere, gaussian or any explicitly given subparticle formfactor[36]. In the same way distributions of particles as e.g. clusters of particles or nanocrystals can be calculated including subparticle asymmetry and random position fluctuations by a Gaussian distribution as Debye-Waller factor (see structure factors). In addition, the asymmetry factor of the particle is calculated to be included as a correction for the structure factor[37]. Oriented particles can be simulated by *orientedCloudScattering* limiting the orientational average to an oriented cone and calculating a 2D scattering pattern. On one hand, the resolution of a subparticle grid is a kind of volume integration over the particle that needs finer grains if the resolution is increased. On the other hand, the substructure of any particle lattice leading to Bragg peaks is observed (like atoms in a crystal). This allows to examine the crossover from particle shape scattering to internal structure as shown later in a structure factor example.

Methods to build clouds of scatterers e.g. a cube decorated with spheres at the corners can be found in Examples module.

### Structure factors

The module *structurefactor* (shortcut *sf*) contains several structure factors for crystals with a long-range order and for particle suspensions without long range order. Structure factors for crystals with cubic symmetry (sc, bcc, fcc), diamond lattice, hexagonal lattice (hcp, hex) or general rhombic lattices with multi atom unit cells can be calculated. Bragg peak broadening due to limited domain size [38], peak asymmetry, Debye-Waller factor and asymmetry of the particles[37] can be included in the structure factor as described extensively by Förster[39].

**Fig 4:**
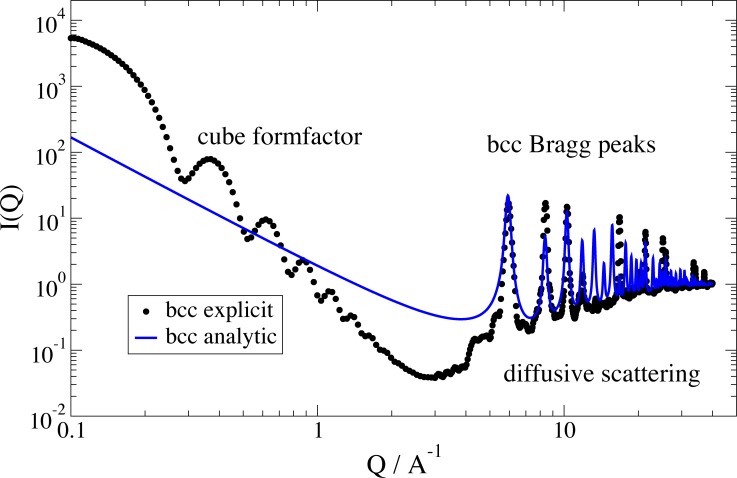
Comparison of analytical structure factor model with an explicit calculation for a bcc lattice in cubic shape of same dimension. At Q = 5 A^-1^we observe the onset of the (100) peak of the respective simple cubic lattice, which is forbidden in a bcc lattice. At low Q we observe the form factor of the crystal shape as a cube.

**Fig 5:**
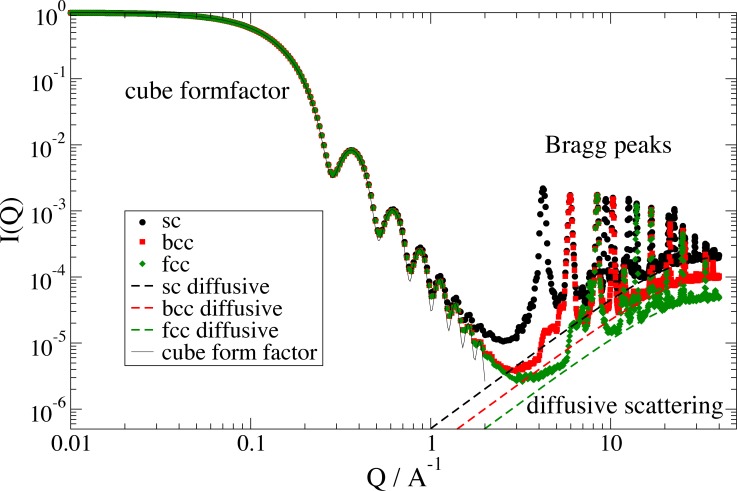
Simple cubic (sc), body centered cubic (bcc) and face centered cubic (fcc) lattices in a cubic cluster shape. At low Q the analytic form factor of a cube is shown. The explicit calculation shows a reduction of the peak intensities due to the Debye-Waller factor, which additional causes diffusive scattering at higher Q. Because of the incomplete lattice planes the extinction rules of fcc and bcc lattice are not fulfilled completely resulting in additional small peaks at forbidden peak positions below the first regular Bragg peak. Calculated within example 18 in Jscatter.

As the previous analytical treatment of the lattice structure factor does not account for incomplete unit cells or arbitrary lattice shapes (e.g. for spherical or cubic nanoparticles) and does not represent the low Q behavior satisfactorily (see discussion in [39]) the explicit calculation from a grid of particles by explicit calculation allows to compute the structure factor of arbitrary shaped clusters. Therefore an explicit grid with the desired geometry is constructed and the function *ff*.*cloudScattering* is used to calculate the structure factor. Examples for cubic lattices with a comparison to the analytical model are shown in Figs 4 and 5.

Non-crystalline structure factors are derived from the pair interaction potential between particles. The simplest model results from the hard-core potential represented in the Percus-Yevick structure factor in 3D[40,41]. This potential is additionally given for the case of 2 and 1 dimensional problems[42,43]. An attractive interaction with a hard core can in the simplest case be represented by a potential well in the sticky hard sphere or adhesive hard sphere structure factor [44,45]. The structure factor of a critical system is described by Chen as calculated in *criticalSystem* [46].

**Fig 6:**
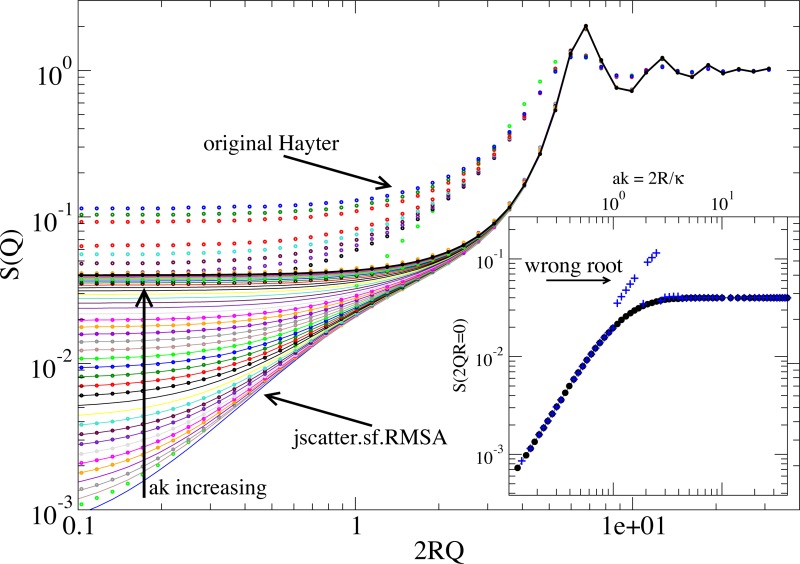
Comparison of the original algorithm of Hayter (dots) with the improved algorithm selecting the best solution of all 4 roots in Fw(w_i_) (lines) for a contact potential Γ = 3 kT, volume fraction Φ = 0.4 and dimensionless screening *ak* = 0.1–58. We observe the second set of solutions representing the wrong solutions (only dots) and some solutions missing in the original solution (only line). The inset shows the respective S(Q = 0) values for the improved solution (black) compared to the original solution (blue). Here some points are missing as no solution was returned others show the wrong solution.

The interaction potential between charged spheres with a screening due to an ionic solvent is described by the repulsive screened Coulomb pair potential. The resulting structure factor in rescaled mean spherical approximation (RMSA) was original published by Hansen and Hayter[47] and Hayter released an algorithm in Fortran 77 (1981, ILL Grenoble). Today most programs implement code directly derived from the original code translated to C or other languages. The rescaling of the MSA solution is necessary as it yields a negative value for the radial distribution function g(r) at r = R at low volume fractions [47]. The Python code here is also derived from the original Hayter Fortran code with an important deviation. The original algorithm determines the root of a quartic Fw(w_1_,w_2_,w_3_,w_4_) by an estimate (named “P-W estimate” in the source code), refining the estimate by a Newton algorithm to find one of the central roots of 4 roots. Dependent on the used parameters, the “P-W estimate” is not good enough resulting in an arbitrary root of the quartic in the Newton algorithm. This results in the correct solution, a solution with g(r<R)≠0 or no solution. Fig 6 shows exemplary a comparison for Γ = 3, R = 3.1, Φ = 0.4 and 0.1<ak<58. We apply here the original idea from Hayter[47] to calculate G(r<0) for all four roots of Fw(w_1_,w_2_,w_3_,w_4_) and select the physical solution with g(r<R) = 0. The roots are directly calculated by *numpy*.*roots* determining the eigenvalues of the companion matrix[48] and g(r) is calculated by the sin-transform. Because of the inversion problem related to a limited Q range and number of points[49], the solution with a minimal g(r≈R/2) is chosen (see Fig 6 lines). The shoulder observed for small ak around 2QR = 1 in Fig 6 is already described by Hansen and represents the change from the long range repulsion to the short range hard core repulsion[47]. The second set of solutions with too high structure factor values at low Q represent the unphysical solution due to the wrong root.

The hydrodynamic function H(Q) describes the hydrodynamic pair interaction between spherical particles in solution for finite concentrations. It is required within a correction of the observed collective translational diffusion coefficient D_eff_ from the single particle translational diffusion coefficient D_0_ as D_eff_(Q) = D_0_H(Q)/S(Q) with the structure factor S(Q) [50,51]. The correction can also be applied to describe the translational diffusion of rigid proteins at finite concentrations[52]. We apply the theory from Beenakker and Mazur as given by Genz and Klein to calculate the δγ-expansion for many body hydrodynamic interaction within a renormalization approach [53–55]. Within the δγ–expansion the hydrodynamic function H(Q) can be calculated based on a structure factor S(q). Additionally, the self-diffusion coefficient D_S_ is calculated. For a description of the function see Genz and Klein for details[55].

### Dynamic

This module contains various models describing dynamic processes mainly used in context of inelastic neutron scattering to describe backscattering, time of flight experiments (BS, TOF, both measure in the frequency domain) or neutron spinecho spectroscopy (NSE, measures in time domain). Models describe generally the intermediate scattering function I(q,t) in the time domain or the dynamic structure factor S(Q,w) as the Fourier transform of the previous. The Fourier transform is implemented in the function *time2frequencyFF* from time domain to frequency domain. The advantage of the time domain is that the combination of different processes is done by multiplication, including instrument resolution. In the frequency domain this is realized by a convolution, which needs more computing time. A function for the binning in frequency intervals is given to implement averaging over different channels.

Common models in the time domain include simple diffusion, stretched exponential, jump diffusion or methyl rotation [56,57]. Diffusion in a harmonic potential for 1,2 and 3 dimensions is implemented[58]. *diffusionPeriodicPotential* describes fractal diffusion with a fast in trap diffusion and a long time diffusion in periodic potentials[59].

Finite Rouse and Zimm model for polymers including internal friction[60–62], the Zilmann-Granek model for bicontinuous and lamellar emulsions for coherent scattering are implemented[63]. Rotational diffusion of an object like a protein described as a cloud of scatterers can be computed[64,65].

**Fig 7:**
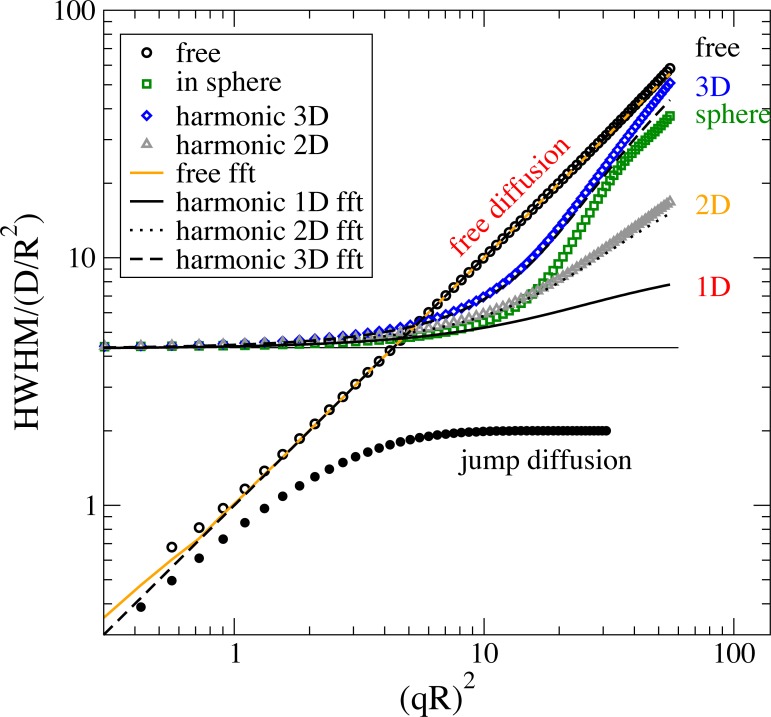
Half width half maximum (HWHM) determined from various dynamic models in comparison. Points show models in frequency domain as indicated. Data resulting from time domain models using Fourier transform are shown by lines. The plateau at low QR values demonstrates the effect from spatial restriction. At high Q a slope dependent on the dimensionality of the diffusion type is observed. The model for 1D diffusion in a frequency model is missing as the corresponding function in the reference seems to be wrong[58]. At very low QR determination of HWHM gets inaccurate. Calculated by Example 12 in Jscatter.

In the frequency domain the diffusion in a sphere, diffusion in a harmonic potential, rotational diffusion and n-site jump diffusion are implemented additional to elastic scattering, translational diffusion and jump diffusion[58,64,66,67]. Fig 7 shows a comparison of the half width at half maximum (HWHM) for different diffusion processes computed in the frequency domain and in the time domain with FFT to frequency domain.

As an example, we may look at the dynamics expected for a protein in solution with rotational and translational diffusion and additional internal mobility of protons in a harmonic potential. The model is similar to the investigation of alcohol dehydrogenase in solution by Monkenbusch et al. It was found that protons close to the surface show a fast localized diffusion[68]. The dynamic structure factor *S*(*ω*,*Q*) can be described by the convolution of the respective processes:

$$S_{total}(\omega ,Q)=S_{tranlation}(\omega ,Q)⊗S_{rotation}(\omega ,Q)⊗\left((1-f_{surf})+f_{surf}S_{harmonic}(\omega ,Q)\right)$$

**Fig 8:**
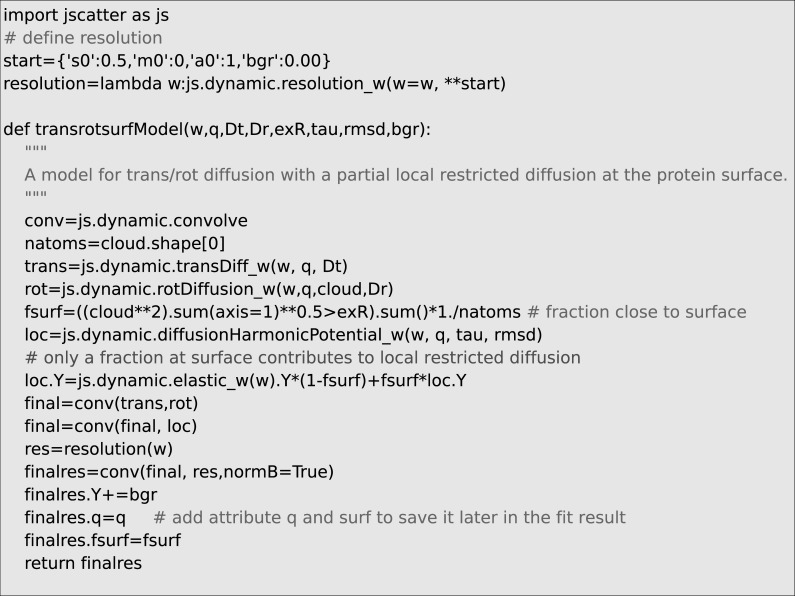
A script snippet showing how to define a function for fitting. Here the model includes a cloud of points describing amino acid positions in Ribonuclease A, translational and rotational diffusion and the diffusion in a harmonic potential for a fraction of the surface amino acids. The resolution may depend on Q. Alternatively a resolution measurement can be used. The full example script is shown in Jscatter module *examples* including reading of the corresponding protein structure file saving the protein coordinates in *cloud*.

**Fig 9:**
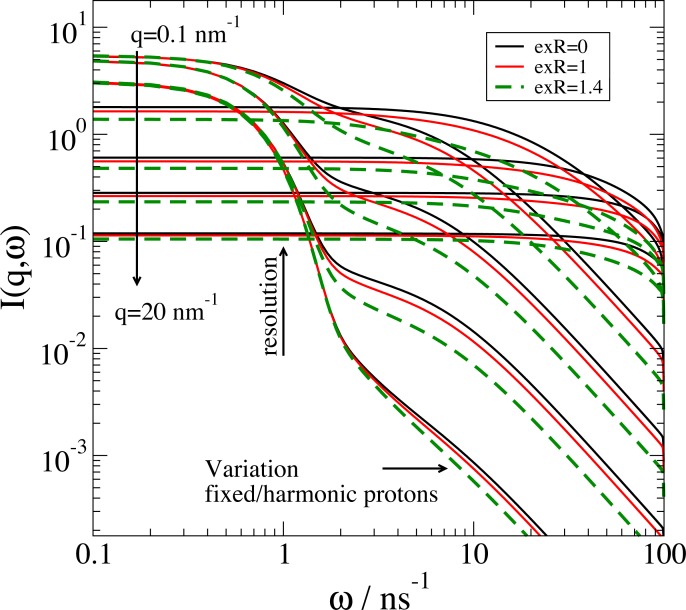
Dynamic structure factor for restricted harmonic proton motion at the surface of ribonuclease A with contribution from translational and rotational diffusion as calculated from the model as defined in Fig 8. We observe a characteristic change in intensities if the fraction of mobile protons is more reduced to the surface protons with increasing *exR* as the radius from the center of mass with fixed protons. The resolution width is 1 ns^-1^. The diffusion coefficients correspond to the expected values for Ribonuclease A in a D_2_O buffer at 20° C. Calculated by Example 13 in Jscatter.

The restricted motion in the harmonic potential may be added only to the fraction of protons f_surf_ that are close to the surface of the protein. The protein general shape is reconstructed from the C_alpha_ atoms in the atomic structure from ribonuclease A (entry 3rn3 in protein data bank, PDB) with the assumption that all amino acids scatter in a similar way as protons dominate the incoherent scattering. The proton surface fraction is approximated as fraction outside of a distance from the center of mass for the globular Ribonuclease A. The definition of the corresponding model is shown in Fig 8 and the results is shown in Fig 9.

### Examples

Models and functions contain an Example section in the documentation that shows basic usage and explains the parameters. Additionally, the module *examples* shows use-cases to allow easy adaption for the user. For example “*Analyse SAS data*” explains how to extract form and structure factor from a concentration series in small angle scattering by extrapolating to zero concentration. Examples are provided as scripts including example data to allow direct execution and inspection of the results. They allow to simulate experiments as in the previous example to test which concentrations are needed for a good extrapolation to zero concentration. Included are examples that demonstrate basic usage of Jscatter e.g. how to build simple and more complex models, smoothing of X-ray data, how to include resolution smearing for SANS data or diffusion of proteins in solution to demonstrate fitting to a diffusion model. These examples show how to plot and the basic capabilities. Sinusoidal fits and multishell cylinder models present fit capabilities by the Levenberg-Marquardt fit algorithm. Smearing and desmearing of SAXS and SANS data is demonstrated. Different models are shown to describe the variety of samples that might be described by the models as e.g. multilamellar vesicles. The examples will be extended to more use cases. Most of the examples with corresponding figures are included in the Examples section of the online documentation.

## Requirements/Extending

The most common libraries for scientific computing in Python are NumPy and SciPy. These are the only obligatory dependencies for Jscatter beside matplotlib for plotting and Pillow for reading of images. Python in combination with NumPy can be quite fast if the *ndarrays* methods are used consequently instead of explicit for loops as NumPy methods use compiled code. E.g. the *numpy*.*einsum* function immediately uses compiled C to do the computation. SciPy offers mathematical functions, e.g. optimization, special function or quadrature, and optimized algorithms also from blas/lapack. For advanced users common packages as Numba or computation on Graphic card can be integrated within user-supplied model functions. As these are more specialized and not easy to implement for most users they are currently not described. Speeding up Jscatter by Fortran code is applied in the function *ff*.*cloudscattering* whereas prerequisite the gfortran compiler is needed which is common on Unix-like systems. The Python interface to compiled Fortran code is automatically generated by *f2py* (a part of NumPy) if Fortran90 code is placed in the specified folder of Jscatter. Using OpenMP, an API that supports multi-platform shared-memory multiprocessing (*www*.*openmp*.*org*), inside of the Fortran code allows usage of shared memory and multiprocessing reaching the advantages of pure compiled code on multi CPU machines. Fortran usage is explained in the documentation and used in *ff*.*cloudscattering*. The speed up compared to compiled C code implemented in Numpy (e.g. *numpy*.*einsum*) is by a factor of 6 in the used example.

Users can write their own modules and import them in Python. Contribution of modules or single models is welcome and can be incorporated in Jscatter or published as separate package importing Jscatter.

## Perspectives

Jscatter implements a data structure with metadata access to allow users data treatment, model building and fitting in a simple fashion using an open scripting language without the need of deep knowledge of programming languages. An extensible environment with a model library currently focused on scattering is provided. Additional to the implemented models more form factors will be included and the *structurefactor* module will be extended to allow more complex structure factors as multi Yukawa potentials[69,70].

In the tradition of utilization of Python as a glue[71] additional capabilities based on external open projects will be added. E.g. Bayesian analysis as used for SAS or DLS analysis would enhance the optimization capabilities beyond Χ^2^-minimazation and provide an alternative to the CONTIN algorithm in the *dls* module [72]. To allow modelling of structure and dynamics of proteins with atomic detail e.g. from PDB data bank or MD simulations with respect to scattering measurements a module for handling atomic PDB structures will be included[73]. Simplified interfaces as software wrapper to well-known software in Fortran (like the *jscatter*.*dls* module for the CONTIN algorithm) may provide an opportunity for less advanced users and integrate well developed software in a joined workflow. Additional examples will illustrate how to use external libraries in addition to Jscatter to read common file formats. E.g. the NeXus format widespread in neutron and X-ray scattering may be read using the *NeXpy* library and transferred to a *dataArray/dataList* including metadata for fitting[74,75].

Designed as an open source project contribution of models, new topics or tasks (e.g. coarse grain simulation) are welcome and will be included into Jscatter to extend the covered scientific areas and techniques.
